# Ergot; Its Natural History and Uses as a Therapeutic Agent

**Published:** 1861-02

**Authors:** E. N. Chapman

**Affiliations:** New York; Professor of Materia Medica and Therapeutics in the Long Island College Hospital


### SELECTIONS.

*Ergot; its Nrtural History and Uses as a Therapeutic Agent.* By E. N. Chapman, M. D., of New York. Professor of Materia Medica and Therapeutics in the Long Island College Hospital.—Ergot (*Secale Cornutum*) is defined to be the disease seed of the secale cereale or common rye. Though more frequently found on this plant, yet others of the same family, the grass tribe—gramineæ—are often thus affected. In the wheat, (triticum vulgare,) for example, the spur is common, and it has been shown by the observations and experiments of Mialhe and Pareira to have the same chemical constituents and to produce a like action on the human organism. This parasitie growth possesses undoubtedly a fixed, established character on whatever plants it may be developed, not only when appearing in the grasses, where it has been observed in thirty-one species, but, also, when attacking the sedge family—the cyperaceæ—to which it is less natural. We know this must be so, from whatever source the ergot draws its nourishment, since, from recent observations, it has been established that this and all other fungi have laws of development as unvarying as those that preside over the growth of a tree or animal. The evolution of its germs, like every other form of life, is dependent on a suitable nidus, which can afford the materials for its increase. This is presented by the ova of the grain with its store of albuminoid products. These, elaborated by the plant for the perfection of the grain, are attacked by this parasite, and so transformed by a special, vital powder, that every vestige of original conformation disappears, and a new vegetative product takes its place. This product is the subject of our present observations.

It is called ergot, a name in the French for the spur of the cock; secale cornatum, horned rye, in the Latin; and Mutterkorn, or wombgrain, in the German languages; the two former names originating from its form; the last from the popular belief as to its virtues. Diverse opinions have been entertained as to its nature and mode of production.

Some regard it as a diseased condition of the seeds of the grain produced by the attacks of insects, or seeds perverted in their growth by climatic influences, as, for instance, excessive moisture conjoined with high heat.

A second view is that the ergot is a fungus, growing in the glumes of grasses in place of the ova of the grain, which it entirely supplants. De Caudolle, as early as 1855, and Guibourt, in 1849, both distinguished observers, adopt this explanation.

A third view combines the two preceding, and supposes that the spurred rye is the combined product of the morbid grain and the fungus, that its power as a medicine arises from this union, but which of the two was the most important was not decided. Quekett, Leveille, Phillipar, and others advocate this hypothesis, in which they are followed by Pereira, Wood, and most other writers. Mr. Quekett, by extended observations, showed that a fungus, the ergotaetia abortans, or oidium abortifaciens, first makes its appearance on the young grain in the form of a mildew, which, under the microscope, is found to consist of an infinite multitude of spores, which rapidly shoot into cobweb-like filaments, interlacing in all directions, and cementing anthers and stigmas together. This fungus was thought to be mingled with the remains of the decayed seed and the product to form the substance called ergot. Now, from the most recent investigations in vegetable physiology, we must arrive at the conclusion that the fungus, and the fungus alone, is the only indispensible and special constituent of ergot. Remove this, and we have no proof that any other state of disease in the grass tribe will cause a morbid product that possesses the toxic and medicinal action of this drug. We know that the bloom of spores on the ergot, appearing like a fine dust, when sprinkled on the flowering grasses or at their roots, will produce the parasite, and that seeds immersed in water containing them become ergotted. We know that the ova perish, literally starved, and that all of their store of albumenoid products, which naturally would be elaborated into starch granules, is appropriated in the nourishment of a parasite, which, by a vital power of its own, transforms starch into oil, and from a nutritious substance prepares a poison that mysteriously causes contraction in the uterus, and, long used, mortification of the extremeties. We know that no trace of starch can be detected by iodine in this new substance, as there would be, were there any of the grain remaining unchanged.

This point is rendered still more certain by the discoveries recently made and established in regard to the laws of reproduction and growth of the humblest cellular plant, or even a plant of a single cell of microscopic minuteness, that are found to be as fixed as those regulating the life of higher organizations. In fact, all life seems to begin on the same level, only differing in the extent of its development, and the germ cells that eventuate in a human being pass first through all the stages of those of a plant. By the latest observations of Kolliker, in his recent work, it appears that the male germ originates in the testis as a globular cell with a cell-wall, containing from one to twenty nuclei, and that these nuclei are the heads of the spermatozoa, which are folded up in the cell. This cell-wall bursts by the time it reaches the rete testes and the spermatozoa, which are nothing less than elongated cells, are set free. I should state that this high authority has proven that the nucleus in all cells is likewise a cell with cell-walls containing a nucleus, (nucleolus,) and that this nucleolus is supposed by him to be constituted in the same way. The female germ-cell, in as far as can be discovered, is identical with all other cells wherever found, and has a striking analogy to that from the testis. The cell-membrane is named vitellin, the nucleus, germinal spot. In fact, there is no essential difference in any cells, whether of growth, exertion, secretion, or re-production. All have a cell wall, nuclei and nucleoli, and contain in their structure, especially in the cell-wall and nuclei, a protein substance that is universally present.

In reproduction the contents of the male and female cells mingle, and, at the present time, this mode of propagation is being established as the universal law of nature for every living thing. As late as 1855 and 1856, it has been satisfactorily demonstrated by Cohn* and Pringsheim† that the spores of fungi are fertalized in a mode precisely similar to that before known in regard to the ova of animals and flowering plants. The spermatozoids, first made known by these two writers, are proven to be strictly counterparts to the spermatozoa of animals and the pollen of plants. Universally the male cell has one or more filament-like projections in ceaseless motion, and this has been shown to enter the female cell wall either by its absorption or by the law of endosmosis. Then begin those changes that in one case form spores, in another a tree, in another an animal, and in another a man. Each starts from

* In Comptes Rendus, vol, 43, 1856, and Ann. Sci. Nat. Ser. 4 vol. 5, p. 323.

† Royal Academy of Science, Berlin, Ma., 1855, and May 1856 and Ann. Sci. Nat, Ser. 4 vol. 8, p. 363, and vol. 5, p. 250.

the same point—cells containing or their nuclei and walls being formed of, nitrogenized principles—and by a life-power each individual is envolved according to his kind—first, however, passing through the stages of the inferior orders. To illustrate, the fungi belong to the lowest order of plants, the cryptogamous. Of these, on the verge of vitality, are those of a single cell, either globular or elongated; an example of which is afforded by the green scum on standing pools. A modification of this form of development is seen where the cells elongate to end in jointed filament-like prolongations, as in ordinary moulds or mildew. There is the bread mould and the cheese mould living on dead organic matter, and moulds nourished by the juices of living plants, as that causing the rot in potatoes, or that which has, for several years, destroyed the grape crop of Europe. The ergotætia abortans or oidium abortifaciens is a fungus of the same simple structure.

Now, it has been proven that all vegetation—the highest and the lowest—is dependent on a vital power seated in the cells. This life-force arises from the protoplasm or azotized principle they contain, and by its presence each cell, individually and independently, effects assimilation and nutrition, and the gross result is but the conjoined product of many cells. By the vitalism of these cells, under the influence of light, inorganic is converted into organic. Carbon, hydrogen, oxygen, and nitrogen are thrown into endless combinations, which chemistry cannot even imitate. The air and soil, gases and minerals are transformed into starch, sugar, acids, oils, vegetable albumen, fibrin, casein, cellulose, lignin, and the thousand other products of the vegetable kingdom. Thus we see that the plant, without a nervous system, affects a higher vital action than the animal, since the latter must have for its nutrition ready prepared and organizable materials, and cannot, like a plant, feed on the elements. The humble spore has a power of elaboration even greater than the human stomach. It changes starch into oil, and the nutritious grain-germ into a powerful poison, and, like every other living being, has a parentage that extends back innumerable generations.

I hope, therefore, that I have sufficiently vindicated the claims of this humble plant to a place in the ranks of the great and ceaseless procession of life that ever is passing over the stage of time.

*Constituents.*—The ergot is covered with a fine dust, which is the sporidia of the fungus. When examined under the microscope, it is found internally to be formed of cells containing oil. After this oil is removed by ether, a substance may be obtained by boiling alcohol, which, by Wiggers, received the name of ergotin, under the supposition that it was the active principle. Bonjean removes the oil in the way mentioned, and from the residue makes a watery extract, which he also calls ergotin. F. L. Winkler obtained a volatile alkaloid, which he called secalin. Probably the active principle has not been isolated; yet, from our present light, I am disposed to conclude the watery preparations may contain it. At least, it is tolerably certain the alcohol is a poorer solvent than water, and that an infusion of fresh ergot rarely disappoints us. There are two principles in this drug; one the narcotic or toxical, which resides in the oil,* as we shall see, and the other the uterine motor-stimulant, which remains in the residue after it has been exhausted by ether.

As early as 1831-2, Dr. Charles Hooker, of New Haven, experimenting with the oil, gave it to a medical Student—ℨ ss. at 2 o'clock; ℨ i. at 3 o'clock, and ℨ i. at 4 o,clock. Intense narcotic symptoms were produced that did not entirely disappear for several days. The pulse fell from 85 to 65, and then to 36, and the respiration from 19 to 8 per minute. On using the same oil, in labor, no influence was observable on the uterine contractions, but on the child that of a powerful narcotic.

He used it in six cases of labor, employing from 20 to 75 drops, and though the pains were not increased, yet "the children for a considerable time after birth had a livid appearance, and respired with much difficulty and irregularty, with the ordinary symptoms of ergotism." In the experiments of Boujean and Wright with the oil on animals, a similar train of narcotic symptoms followed, ending frequently in tonic or clonic convulsions and death. In the many experiments that have been made by others on animals, it has been shown that where abortion does not occur, yet as a general rule, the conception perishes, and after a time is expelled.

Hooker then used the residue that remained after the extraction of the oil, and found its alcoholic power unimpaired. Bonjean's experiments accord with those of Hooker.

We conclude, therefore, that the uterine motor-stimulant power exists in the watery preparations, and the poisonous principle in the oil, but what are the ultimate elements has not been satisfactorily determined.

---

*** It is stated that the oil extracted by ether, on standing, seperates into an upper and lower strata, and that these differ in their properties, the heavy kind causing uterine contraction. The want of proof of any such distinction has led us to pass over this point in silence.**

*History.*—It has been long recognized that a smut or rust in grain has been the cause of epidemics, which in different seasons have committed great ravages in Europe. Formerly more than at present, from the surroundings of the people, filthy modes of living, bad food, the want of vegetables, and defective ventilation in their mud hovels, typhus plagues and spotted fevers were common; but there cannot be a doubt that these were rendered more prevalent and malignant by a diet of ergoted grain. This cause is rendered more palpable by the appearance of gangrene and mortification in many epidemics. The only wonder is, that these poor people, confined almost exclusively to such bread, could, any of them, escape with their lives. It must be, as suggested by Bonjean, that the heat employed in cooking, dissipates more or less the active principles of the ergot.

The oxytoxic powers of this drug have, from time immemorial, been known amongst the common people of Europe. This is shown by its vulgar name Mutterkorn or womb-grain amongst the Germans. The midwives on the Continent employed it so generally in difficult labor that its use was interdicted in France in 1774. Three years after, Dr. Desgranges, of Lyons, introduced the drug into general use amongst the profession, by published reports of its usefulness and efficacy, yet it was forgotton, until its knowledge was revived thirty years subsequently by Dr. Stearns, of Waterford, of this State. Drs. Akerly, Prescott, Dewees, Chapman, Atlee and others, confirmed the report of its power to excite the action of the uterus, and by their countenance to its use in tedious and difficuit labors, rendered its employment in the lying-in room fearfully common. With pride they looked upon it as an American discovery, and as they were enlisted in establishing its claims, and committed to the foregone conclusion that it was a safe and efficacious remedy for expelling the child, they were, of course, blind to the causes of the increased infantile morality.

In France, Chaussier and Madame La Chapelle denied that ergot exercised any power over the uterus. In this they were followed by most of the obstetricians of Europe. Even as late as 1825 it had been scarcely tried, and the unbelief in its power was nearly universal. Even at the present day there is the same doubt, and the properties of our drug are thought to be of a very ambiguous, uncertain character; that, perhaps it may increase the pains when present, or on the eve of arising, but that it cannot originate uterine contractions nor cause abortions.

This is folly. Ergot either has or has not the power over the uterus—it will or will not cause contraction of that viscus when extended.

This we shall discuss by investigating its physiological action.

Animals generally manifest a strong, instinctive repugnance to the ergot, and where considerable quantities are administered, the toxic symptoms are pretty uniform. These are similar to the peculiar condition called ergotism in the human subject. Loss of nutrition, impaired nervous power, imperfect circulation, especially in the extreme capillaries, muscular weakness, sometimes gangrene, convulsions, and death. The pregnant female usually aborted, and where this did not occur immediately, the conception ordinarily perished, and was subsequently expelled.

That the action of this drug is the same on animals as on man, is shown by the experiments of Mr. Tonatt, who says, "I have for the last six or seven years been in the habit of administering the ergot of rye to quadrupeds in cases of difficult or protracted parturition. . . . . The uterus has in every case responded: it has been roused to a greater or less degree of renewed action."

On man, ergot, in full doses, has a special narcotic action, shown by the reduction of the pulse from 10–20 pulsations per minute—giddiness, delirium, dilated pupils, trembling, weakness, unsteady gait, a peculiar intoxication, which the peasants, living on ergoted grain, seem to enjoy, as they would the excitement from alcohol or opium. By living on contaminated food for a length of time, the mind becomes dull and imbecile. There is formication, numbness, cramps, cold and livid extremities. Sometimes convulsions, and speedily mortification, which commences in the extremities and passes upward. It appears as though the narcotic paralyzed the nervous system, rendering the capillary circulation feeble at first, and then finally checking it altogether. Hence, the extreme vessels collapse and dry gangrene is the result. On this property is based the use of this drug in any of the different forms of hemorrhage. Continued doses are required for some time, and a near approach at least to this peculiar ergotic condition. But as we have a safe and far more reliable remedy in the persulphate of iron, or gallic acid, we scarcely should be justified in using so hazardous an article as this would be in persistent doses.

The second property of ergot, and the one for which it is mostly used in medicine, is the power which it has of contraction of the muscular fibres of the uterus. This effect follows, generally, within the hour, from small doses of from 20 to 40 grains, quantities that would occasion no symptoms of ergotism, or any of depression of the heart and nervous system.

*Mode of Operating.*—The ergot, like all systemic remedies, is absorbed, and thus, through the circulation, impresses the motor tract of the spinal marrow in that limited portion, where-ever the uterine nerves take their origin. The muscular organs, contiguous and supplied by the same nerves, as the bladder, for instance, are, as it has been shown, subject to the same stimulus. Herein is comprised all of the valuable virtues of this drug. It is simply an excitant of the motor nerves of a limited portion of the body.

Strychnia is analogous in its mode of operating, affecting, however, the entire motor tract of the spinal marrow, but having no influences over the nerves of sensation or the brain. Both of these drugs act centrically, and thus arouse those reflex movements, of which the spine is the special seat.

The peculiar phenomena excited by ergot have been mostly studied in connection with its medicinal employment. In from 15 to 30 minutes after exhibiting a full dose to a woman in labor, the pains become greatly intensified and without intermissions, although there are remissions for a few moments, and then exacerbations. The womb is constantly hard like a stone, and at no time relaxes. The child is crushed firmly down, does not recede as in ordinary labor, and all the soft parts of the mother are subjected to an unremitting pressure, which in the exacerbations tells with fearful energy on both mother and child. There is no peace until either the child is born or the excitability of the uterus is exhausted. After labor, the uterus condenses into a hard globe, and does not have alternate periods of contraction and relaxation. That good ergot will produce this result with great uniformity I am thoroughly convinced, from a considerable experience. It is alleged by those who grant the power of the drug to increase the natural pains or excite them, when on the eve of arising at the full period, that at no time antecedent has it any such power, and that it cannot originate labor per se. This is an important question practically, and in a medico-legal point of view. During gestation, the uterine muscular fibres are increased many hundred times, and the nervous and vascular supplies are equally augmented. The fully-developed organ would feel any special stimulus more sensibly than at any previous stage, when the instruments of its appreciation are less. Obviously the nearer the uterus is to its normal size, the less likely shall we be able to arouse it to contraction, and under the same circumstances, we shall be less likely to succeed in morbid distentions than in pregnancy, since in the latter case, it is in a high state of vital activity, whereas when a polypus occupies its cavity the muscular fibres are merely stretched asunder by an internal force. From my experience, I am certain that ergot has a direct power to excite the contractions of the uterus at all times when distended. In some most desparate case of flooding after delivery, where I had extreme difficulty in staying the flow until the medicine could be procured, I have seen it in fifteen minutes produce a firm condensation of the womb, and I have left my patient with a perfect confidence that the danger from this source could not arise again.

A lady, the mother of several children, and of a delicate, spare habit of body, was taken with the most alarming hemorrhage within an hour after delivery. The labor was rapid, with only two expulsive pains. Everything was drenched with blood. It was found that the womb would not contract on the hand in its cavity, and could only be imperfectly retained in a semi-firm state by ice in the vagina, friction with ice externally, and strong compression. She passed from one fainting fit to another, in spite of brandy, which seemed to produce little or no stimulation, and, beside, it was impossible completely to stay the flow that was adding to the collapse. Two drachms of powdered ergot had no effect. At the end of two hours matters had but little mended for the better; the chief change was occasioned by a slight rallying from the stimulus of the brandy. A fresh specimen of ergot was now procured and administered, and in twenty minutes, after giving an infusion of twenty grains, the womb began to condense its fibres, and in less than an hour, by repeating the doses, the uterine globe was firm and hard, with no alternate contractions or relaxations. In another case, resembling this in every particular, the ergot had the same effect.

In both, the exhaustion was so great as to render the response to stimuli imperfect and scarcely perceptible, although given with a liberal hand; yet the ergot, in spite of the want of a disposition in the uterus to contract, caused it to do so within twenty minutes, and not only this, but rendered its condensation firm and unremitting. Surely there cannot be a doubt of a remedy that acts on the nervous centres when brandy produces little more effect than so much water.

In several instances where, from the death of the conception or the alarming loss of blood on the part of the mother, it has been necessary to hasten the miscarriage, I have rarely been disappointed in this drug. In one case I succeeded with it, where from a fœtus in a putrid condition, the gravest typhoid symptoms had arisen, as shown by a pulse of 140 beats per minute, a dusky countenance, and a low muttering delirium. The lady was advanced to about the fourth month, and although the conception had probably been dead for three weeks, as at that time she fell insensible in puerperal convulsions, yet there was no indisposition to contraction in the uterus. The first prescription failed, but on the next day, the ergot was produced from a sample which I knew was good from previous trial. Within an hour its peculiar action was manifested, and this was continued by repeated doses until the fœtus was expelled.

I saw a case with my friend Dr. Davol, which was even more remarkable. A lady, four months pregnant, had become reduced to such a condition of vital prostration and inanition by persistent vomiting and purging, that her case had been pronounced by us and another practitioner a hopeless one. There did not seem to be any chance for the induction of premature labor. After continuing in this way for three weeks, first the vomiting and then the purging abated. She seemed to rally considerably; took beef tea, stimulants, &c. Improving slightly, she was taken with uterine pains, which were, however, only momentary twinges in the back for 15 or 20 seconds at a time, recurring about every hour. The typhoid state was extreme as shown by her pulse, the want of response to the stimulus of brandy in ounce doses, the ecthyma covering her body, and a bed sore as large as a quarter of a dollar over the sacrum. The os uteri was dilated, yet there was not vital energy sufficient to arouse the uterus into action. For eight hours she took a preparatien of ergot with no effect. We then procured another known to be good, as we had before used from the same parcel. In fifteen minutes, after taking twenty grains, she had a marked pain, which continuing three or four minutes, forced the uterus low down into the excavation. From the want of excitability in the nervous system the pain went off, but recurred again in the same manner from a fresh dose. The lady was delivered one hour and a half after the induction of the first ergotic pain. In neither of these two cases, both of which took four drachms of ergot by infusion, were any of the narcotic effects of this drug observable, although the feeble condition of the patient was such as, in the opinion of many, to interdict its use.

I saw recently, with my friend, Dr. Gilfillan, a case of polypus uteri, where, in 48 hours, two drachms of the ergot was given in substance. At the end of this time, it was found that no pains had been excited, but the pulse was reduced from seventy-four to sixty-five beats per minute, and there was experienced a dizziness, heaviness in the head, and an uncertainty in muscular movements, showing conclusively that the nervous centres were affected. As the quality of the sample of medicine we were now using was evidently deteriorated, it was changed for a fluid extract, made from the ergot employed in the last two cases before mentioned. Of this there was administered fifteen minims, (the extract containing one grain to the minim) every five hours, until one ounce and a half was taken. From fifteen to twenty minutes after each dose, the pain would commence, lasting for about two hours, and having that constant tension so characteristic of ergot, when gradually it would subside, as the nervous excitability became exhausted. No effect on the pulse or other symptoms of ergotism were observable, notwithstanding the use of so large a quantity of the drug,—sufficient, had the poisonous principle still remained in it, to have produced the most serious consequences. Before attempting the removal of the polypus, whose pedible was attached to the fundus, it was thought that a dose of the medicine would bring the growth more in reach, and thus facilitate its removal. On examination we found the uterus relaxed, the neck soft and dilated, with no periods of contraction, and, in fact, the patient had been free from pain for several hours. The doctor, who very neatly and skillfully removed the polpyus by torsion, stated that the body and neck of the uterus condensed themselves so firmly, that little space was allowed for manipulation, and his fingers became numb from the pressure. In his opinion there could not be a doubt —in this case at least—of the direct and positive action of the ergot.

*Therapeutical Uses.*—The employment of ergot in labor has been frightfully common in this country, arising, probably, from the fact that high American authorities recommended it as an expulsive agent, both to arouse the uterus when exhausted, and to increase its energy when its efforts, however vigorous, were insufficient. As in the normal process the child could not be born without pains, it was a natural conclusion that these, when feeble, flagging, or incompetent to overcome the resistance, must be stimulated until our great object, the birth of the child, was attained. It is a *reasonable* doctrine that ministers to a convenient and seductive practice, gratifies our propensity to do something, contributes to our ease, and will undoubtedly save us several hours patient waiting. Of course, its advocates, fortified by a wonderful light from reason in the closet, never saw any harm resultiog from it. They do not believe it would injure either mother or child, as though their medical creed made any difference with facts.

It was found, by M. Depaul, that, during active natural pains, the fœtal circulation became greatly hurried, and at times could not be heard; but, during the intermissions, it gradually resumed its normal rythm. This is readily explained by the fact, that the child's blood is aerated through the influences of the mother's in the uterine sinuses, and the circulation in these sinuses and the arteries that supply them cannot go on freely when the muscular fibres are condensed. How much greater will be the danger to the child when, by ergot, the natural pains, are greatly intensified and the contraction is rendered permanent and continuous? The truth of this is rendered evident by statistics.

In 1850, the Academy of Medicine, at Paris, directed to investigate the influence of ergot on the life of the child, come to the conclusion that this was endangered both by compression and narcotism; in 1853, the Academy adopted the conclusions of M. Depaul: "that, except in miscarriage, in certain labors attended with hemorrhage, and occasionally at the conclusion of natural labor, paturient women would be the gainers by the complete disuse of ergot."

In one hundred and seventy-five cases reported by Busch, where ergot was given, seventeen children were born dead, and eight asphyxiated; of eighty, by Chatto, ten were dead; of forty-eight, by Hardy, thirty-four were dead; of sixty-four by R. U. West, nine were dead.

Dr. Stearns, who re-introduced this remedy into notice, and used it more cautiously than many do at the present day, lost his practice from a strange mortality amongst the children and a child-bed fever that followed him, like an evil genius, from door to door. There can scarcely be a doubt that one hour's ergot pain would be fatal to one half of the infants. They are literally smothered, since they are effectually shut out from the only source whence their blood could be supplied with oxygen. I should not deem it safe to administer the ergot in any case, unless I was quite certain that the child would be born in four or five vigorous pains. If, after giving it, the birth should be delayed more than half an hour, it would be more prudent to conclude the labor with forceps.

We should never use ergot as an expulsive agent—never where the pains have been, or are vigorous—never to overcome impediments, as a narrow pelvis or rigid perineum—and never in a primipara under any circumstances. In cases where the pelvis is large, presentation natural, all the parts relaxed, and the previous labors short, we should give it, if from a sudden inertion of the womb the child is left pressing on the floor of the pelvis, partially out of the womb. It might not be safe for the child or the mother to allow it to remain any time in this position, since the pressure might endanger the mother's soft parts, and as, more especially, the child might perish from a partial detachment of the placenta, or obstruction to the circulation in the cord. Either the ergot or the forceps will be required. Should the case appear to require the instruments, the ergot may still be requisite after delivery, to prevent post partum hemorrhage.

*Flooding after Delivery.*—In patients who almost invariably flood after the birth of each child—in all hemorrhage after delivery where the uterus is flabby and relaxed—the ergot is of inestimable value. It will, with great certainty, insure a firm contraction. In any case where we learn the patient floods after confinement, we should give the ergot befo e we leave the house.

*Retained Placenta*—Some give this drug to remove the placenta when retained. A moment's reflection will convince us that this is a most uunjustifiable practice. We have seen that the ergot influence causes a tonic, unrelenting spasmodic contraction of the uterus far exceeding the natural pains. We know that where the drug is not given, it is a difficult matter, two or three hours after delivery, to introduce the hand and remove the placenta. It would be scarcely possible to do so, if this natural condensation was rendered sudden and permanent by this potent agent. Ergot could not displace an adherent placenta—might not overcome an hour glass contraction; and, in the event of its failure, the difficulties of the case would be greatly augmented.

*After Pains.*—The after pains are often rendered more troublesome and persistent by a too lax uterus, allowing the blood to ooze freely into its cavity, when, from a diminished

14

irritability, contraction is only aroused by the stimulus of a greater distension. In such cases, where we have considerable show with each pain, I have found the ergot a most efficient remedy. Should the show be slight, and the pains arise more from nervous irritability, opium and camphor are indicated. The ergot would aggravate such a condition. With both too great a show and nervous irritability, ergot and opium conjoined would be found to answer the pupose. The union of these two remedies would not be improper, since the opium acts on the brain, and the ergot on a localized portion of the spinal marrow.

*In Placenta Prœvia.*—Ramsbotham recommends this drug, for the purpose of holding in check any excessive discharge, until the os uteri shall be sufficiently dilated to admit the hand for the purpose of turning. If we have rightly comprehended the action of this ergotic power, the hemorrhage could scarcely fail to be augmented, and the version of the child be far more difficult; perpaps impossible, without undue violence. From the knowledge we have of the styptic virtues of the persulphate of iron, even in the wounding of considerable arteries, it would, undoubtedly, if pressed against the exposed placental vessels, effectually stay the flow of blood for the time. The iron could be easily and thoroughly applied through a speculum by means of lint.

*Ergot in Collapse.*—It is stated by Beatty, Griffin, and Barker that where there are evidences of great depression of the heart and nervous system, as shown by syncope, collapse, and a feeble or scarcely perceptible pulse; the ergot should not be given until this condition has first been removed by the use of the opium. It is asserted that the sedative property of the medicine would tend to prevent reaction, and directly and positively augment the prostration. Now, it is a fortunate circumstance, that as we suppose, the narcotic and ecbolic principles can be separated, and we can produce one effect or the other at our pleasure; since, in debility, we wish to avoid any approach to ergotism, although the emergencies of the case may call imperatively for increased uterine contractions. In no one case have any symptoms of a narcotic action been observed where the infusion, or the fluid extract, have been administered. Hence, we conclude that in these foums we may use this medicine in the most profound collapse, and in any case, as in polypus or hydatids, for many days in succession.

*Hemorrhage during Gestation.*—The ergot could not be admissible at any period during gestation for restraining uterine hemorrhage, since in moderate doses it could only act by causing contraction, and the induction of this condition would be almost sure to produce miscarriage.

*To Induce Premature Labor.*—Good ergot has undoubtedly the power to originate uterine pain, and it has been used for the induction of premature labor in cases where the pelvis is so contracted or deformed as not to admit of the passage of a full-sized child. By the tables of Ramsbotham and Hoffman, it appears that of thirty-eight children, prematurally born under the ergotic influence, twenty-seven perished. This practice should not be imitated where the child is viable, and there is a possibility of saving its life without endangering that of the mother. But, when the death of the conception, or other sufficient reasons, in regard to the safety of the mother, render the removal of the fœtus imperative, then the ergot affords us, probably, the safest means for this purpose, since the pains, from the imperfectly developed muscular structure of the uterus, cannot be excited to a degree that could endanger any of the soft parts of the mother; and more than this, the child's head would be readily moulded into a form suited to the passage.

*Polypi, Hydatids, etc.*—In all cases where the volume of the uterus is increased by any structure loose in its cavity—as hydatids—the ergot should be used for their expulsion, and in the case of polypi, the same remedy affords us the only means we have of bringing the morbid growth through and beyond the os uteri, so as to render its removal by torsion or ligature possible.

*The dangers to the mother* at the full period, from the negligent use of ergot, are a rupture of the body or neck of the uterus, rupture of the perineum, and injury to the bladder. The walls of the uterus at this stage are more than an inch in thickness, forming a hallow muscle equal in tension and cohesion to any other in the body. I can scarcely believe that any medicine, which merely calls out its excitability, can induce such violent contractions as to make a breach in its structure. We have no analogies to lead us to such a conclusion. The most powerful tetanic spasms, whether from disease, or poisonous doses of strychnia, never produce any such result; and where persons have suddenly and powerfully exerted themselves, the tendons have been known to give way, sometimes a few of the muscular fibres, but it must be a rare occurrence for a powerful muscle to be completely torn across. More than this, rupture of the uterus is less frequent in primiparous women, where the resistence would be the greatest. I cannot but think that all cases of this accident in the body or fundus of the womb must arise through a softened condition of its structure from disease, and would occur, whether ergot was given or not; at least, it is well known that most of the cases were in persons with a deformed pelvis, who had, on previous occasions, been delivered with instruments. I have seen four instances of this sad and melancholy conclusions to labor; two in my own practice, and two in that of others. All had been confined from two to four times, and had one or more times been delivered with instruments. All were in the first stage of labor, the os uteri high up, and the pains insignificant and of a cutting character. In none was any medicine given, and in many cases, which happened in my presence, the rent took place during the absence of the pain; in one by sitting up in bed for a drink, and in the other on getting out of bed. In all there was a sharp projection of the promontory of the sacrum, unlike its natural rounded contour, which greatly encroached on the capacity and outline of the superior strait. During the latter months the womb and its contents, no insignificant weight, must have rested on this projecting ledge. The pressure might cause softening, or even death in the tissue, precisely as we observe in bed-sores, or in ulceration of the heel in fractures; more especially would this cause be operative, if the woman by a fall, misstep, or riding on a rough road, should cause the womb to strike forcibly on this projecting point, so as to bruise or cause ecchymosis into its tissue. The subsequent pressure would then be far more likely to cause softening. One of my cases seemed to date from the giving way of a night chair, which brought the woman with force to the floor. A post-mortem could not be obtained, excepting in the case just mentioned, and it was shown on dissection, that the ruptured portion was sphacelated, and near by was another point unbroken, through which the finger readily passed. In this and all the other cases, as was determined on introducing the hand for the purpose of turning, the solution of continuity was on the posterior face of the uterus, at the junction of the neck with the body, in a transverse line, so that the os was partially detached in the form of a ring.

*Rupture of the Neck of the Uterus or the Perineum.*—The conditions under which the neck of the uterus and the perineum are placed are far different. Their tissues must be distended, and fibres frayed out and elongated before the child can pass, and we know, by experience, that alternate periods of tension and rest accomplish this object in the most satisfactory manner; but should the child be pushed suddenly and forcibly forward, a rupture would be very apt to occur just before the dilatation was complete. The rent in the neck will generally commence on its free edge and extend longitudinally, or a rim of the os might be torn off. The capacity for resistance of these parts, thus thinned out cannot be compared to a powerful muscular structure more than an inch in thickness, that condenses itself at each pain.

*The action of Ergot on other Pelvic Muscles.*—If ergot is a motor-stimulant to the uterus, by exciting the spinal marrow at the origin of the nerves of motion in a specified portion, it follows, that other muscular organs contiguous and supplied by the same nerves should be also under the influence of the same remedy. On this ground it may be used in paralysis of the bladder or its neck and spermatorrhœa, where this arises from muscular atony. Dr. Mitchell, one of the council of this Institution, has attained the best results from its employment in this class of cases. Prof. Barker states that he never knew it to fail in retention of urine after labor.

A gentleman, æt. 35 years, had paraplegia on the right side several years since. He has less command of the leg on that side and a partial loss of sensation in the fore-arm and hand. Sometimes, frequent and irresistible calls to pass urine, that cannot be restrained, will torment him for several days; at other times, for many hours, he is in pain from his inability to evacuate his bladder, when suddenly the flow will commence, over which he now has no command. In this case there was evidently a want of muscular power both in the bladder and spincter, alternating with a spasmodic condition. Fifteen drops of the fluid extract was administered four times daily, until one ounce and a half were taken; when the medicine was discontinued, as now his relief from this annoyance was complete. Since his improvement dated from the commencement of this treatment, and nature with her expectant modes had never come to his relief in eight long weeks, the result was ascribed to the peculiar action of ergot. In this case no evidence of a narcotic impression were observable, either by a reduction of the heart's action or unwonted sensations at the nervous centres.

*Ergot in Menorrhagia.*—The ergot has been much given in menorrhagia, and in all other morbid conditions of the uterus, attended with a bloody discharge, as, for example, polypi, fibrous tumors, ulceration of the os uteri, malignant growths, etc. So also in other forms of hemorrhage, hæmoptysis, hæmaturia, et cetera, the same practice is recommended and instituted by several writers. As before remarked, this drug can only act beneficially in arresting any form of internal hemorrhage through its narcotic principle, and to attain this end it must be persevered in until evidences of ergotism are shown in a reduction of the pulse and the supervention of abnormal symptoms in the great ganglionic centres. For this purpose we could not depend upon any preparation deprived of the oil, and in truth the oil would be the one alone fitted to fulfill the indications. Since, however, we are in the possession of more reliable and far less hazardous remedies, we should be scarcely justified in using one that may produce the most lamentable results.

*The Ergot for Female Diseases.*—The ergot has been employed through an obscure idea of its specific virtues for almost every disease peculiar to females; amenorrhœa, leucorrhœa, uterine and vaginal, and gonorrhœa, both of the male and female; yet, we need hardly to remark, there are no known properties in this drug to render it of the least avail in any disease of this character. Since we build our foundations on facts, and have little time for beliefs, opinions, and the dicta of writers, we shall thus briefly dismiss this head of our subject, with the hope, however, that this word specific will ere long be banished from our nomenclature, and that every man in the ranks of our great and noble profession will ever have a reason for the faith that is in him, established on such a basis; notwithstanding it has been scoffed and sneered at as empirical, we shall hold fast to all established truth which now and ever will be found impressed with the seal of Nature, unchanging and eternal.

In conclusion we will briefly recapitulate the main points, deduced from the data hitherto presented.

1st. The active principles of ergot are the products of a a plant, the odium abortifaciens. These are peculiar to it, and are in no wise more dependent on the grain-germ, than any plant is on the soil that affords it nourishment.

2d. These principles—not as yet isolated—are, probably, two in number; one of which, contained in the oil, produces narcotic symptoms, and eventually gangrene; the other, remaining in the residue after the extraction of the oil, acts by stimulation on the motor tract of the spinal marrow at that limited portion whence the uterus and contiguous muscles receive their nerves of motion.

3d. The oil is alone efficacious in any form of hemorrhage, excepting in that from enlarged uterus, and we can only look for favorable results when the system has been brought under its influence, as shown by a narcotic impression on the brain and heart.

4th. So hazardous a treatment cannot be countenanced; since we possess other remedies of known virtues, not only more reliable, but unattended with any risk.

5th. The watery preparations, which, lacking the oil, can be given many days in succession, will act on the motor tract of the spinal marrow, but will not induce ergotism or other evidence of a narcotic poison.

6th. Ergot of good quality causes the uterus to contract with great certainty whenever its cavity is distended, either by a fœtus or a morbid growth. These contractions are tonic and continuous, with periods of remissions and exacerbations.

7th. Hence, this remedy may originate uterine pains at any period of gestation, or cause the expulsion of clots, hydatids, or any other unattached substance.

8th. Likewise, after labor, it will check an excessive flow by closing the mouths of the bleeding vessels through a condensation of the muscular fibres of the uterus. In relaxed conditions it will be, on the same principle, beneficial in afterpains attended with a copious show.

9th. Since we know of no other mode by which ergot can immediately arrest hemorrhage from the pregnant uterus, it should be avoided in all cases where a hope still remains of saving the conception, and its agency invoked only when the fœtus is dead or the safety of the mother becomes our first duty.

10th. In premature labor, induced by ergot, or in labor at the full period, where this drug is given, the child's life is jeopardized in all cases, where for a considerable period previous to the dilivery, the uterus is thrown into violent spasmodic action; since thus the supply of oxygen will be most effectually cut off.

11th. Where the child is viable, the ergot should never be used as an expulsive agent to increase pains, already powerful, or overcome resistance from the soft parts of the mother.

12th. It may be required in cases of inertia of the uterus where, with all the parts relaxed, we are quite certain, both from the present appearances and the history of former labors, that the child would be born in four or five vigorous natural pains.

13th. The dangers to the mother from the use of ergot, arise from the uterus thus stimulated, rupturing the unyielding parts in its transit. Thus the neck of the uterus, the bladder or the perineum may receive serious injury.

14th. Rupture of the uterus could hardly occur, excepting in a diseased organ.

15th. A premature fœtus from its small size and the yielding nature of the cranial bones could scarcely, under any uterine effort, cause damage to the soft parts of the mother.

